# Change in healthcare utilisation after surgical treatment: observational study of routinely collected patient data from primary and secondary care

**DOI:** 10.1016/j.bja.2022.07.012

**Published:** 2022-10-01

**Authors:** Alexander J. Fowler, Bhavi Trivedi, Kambiz Boomla, Rupert Pearse, John Prowle

**Affiliations:** 1 William Harvey Research Institute, London, UK; 2 Barts Health NHS Trust, London, UK; 3 Clinical Effectiveness Group, Queen Mary University of London, London, UK

**Keywords:** anaesthesia, complications, healthcare cost, peri-operative medicine, primary care, surgery

## Abstract

**Background:**

Most patients fully recover after surgery. However, high-risk patients may experience an increased burden of medical disease.

**Methods:**

We performed a prospectively planned analysis of linked routine primary and secondary care data describing adult patients undergoing non-obstetric surgery at four hospitals in East London between January 2012 and January 2017. We categorised patients by 90-day mortality risk using logistic regression modelling. We calculated healthcare contact days per patient year during the 2 yr before and after surgery, and express change using rate ratios (RaR) with 95% confidence intervals.

**Results:**

We included 70 021 patients, aged (mean [standard deviation, sd]) 49.8 (19) yr, with 1238 deaths within 2 yr after surgery (1.8%). Most procedures were elective (51 693, 74.0%), and 20 441 patients (29.1%) were in the most deprived national quintile for social deprivation. Elective patients had 12.7 healthcare contact days per patient year before surgery, increasing to 15.5 days in the 2 yr after surgery (RaR, 1.22 [1.21–1.22]), and those at high-risk of 90-day mortality (11% of population accounting for 80% of all deaths) had the largest increase (37.0 days per patient year before *vs* 60.8 days after surgery; RaR, 1.64 [1.63–1.65]). Emergency patients had greater increases in healthcare burden (13.8 days per patient year before *vs* 24.8 days after surgery; RaR, 1.8 [1.8–1.8]), particularly in high-risk patients (28% of patients accounting for 80% of all deaths by day 90), with 21.6 days per patient year before *vs* 49.2 days after surgery; RaR, 2.28 [2.26–2.29].

**Discussion:**

High-risk patients who survive the immediate perioperative period experience large and persistent increases in healthcare utilisation in the years after surgery. The full implications of this require further study.

**Editor's key points**

• High-risk patients undergoing major surgery are more likely to have ongoing medical problems, but the extent of this is largely unknown.
• This study found that high-risk surgical patients have a large and persistent increase in healthcare utilisation in the 2 yr after surgery.
• High-risk surgical patients are likely to benefit from additional postoperative follow-up and early primary care review after hospital discharge.

More than 5 million surgical procedures are performed in the UK National Helath Service (NHS) each year.^1^^,^^2^ Surgery remains associated with significant short-term mortality; there are about 50 000 deaths within 30 days of a procedure each year. In 2014, 5.1 million procedures took place, and were associated with 49 000 deaths within 30 days.^1^ However, postoperative death only represents the most severe form of harm. Anecdotal evidence suggests that many high-risk patients experience a worsening of their health, developing new long-term disease with a reduced quality of life. There are very few published data describing long-term health outcomes for the overall surgical population.^3^^,^^4^

Previous research suggests that the great majority of postoperative deaths occur in a sub-population undergoing high-risk surgical procedures. High-risk procedures are those with a 30-day risk of death exceeding 1 in 20, which account for around one in six inpatient procedures.^5^^,^^6^ This high-risk group account for 80% of deaths within 30 days after surgery.^5^ High-risk patients are typically older, with a high burden of chronic disease, and require emergency intra-abdominal operations. To date, most research on high-risk patients has focused on risk of death and complications in the immediate postoperative period.^7^^,^^8^ However, the long-term outcomes for high-risk patient groups remain poorly described, but important to understand for patients considering surgical treatments, and those planning their care. Around one in 15 patients require non-elective hospital admission in the year after surgery.^9^ However, this measure overlooks the wider context of healthcare delivery and fails to consider the pre-existing healthcare need patients have before surgery. Most patient care is delivered in the community by primary care physicians, or in hospital outpatient clinics, rather than on an inpatient basis. To date, no study has explored how the trajectory of healthcare use changes before and after surgery among surgical patients or explored the burden of both primary and secondary healthcare contacts.^10^ Burden of healthcare is a measure of days spent in contact with healthcare that reflects illness severity.^11^, ^12^, ^13^ There is evidence that high-risk patients who suffer complications will subsequently experience reduced long-term survival and quality of life.^43^ However, changes in other postoperative outcomes, such as quality of life and burden of healthcare need, are not well described for high-risk surgical patients.

There is a need for evidence describing long-term outcomes for high-risk surgical patients. This would inform decisions made by patients, clinicians, and health policymakers. We undertook a population-level analysis of routine primary and secondary care patient data. Our aim was to describe how the need for healthcare services change in the years after surgical treatment, in particular for patients who survive despite being at high risk of early death after surgery.

## Methods

### Study design

This was a prospectively planned analysis of routine primary and secondary care data describing patients undergoing non-obstetric surgery within the four surgical hospitals at Barts Health NHS Trust who were registered with a general practitioner (GP) in a local clinical commissioning group (CCG) area. Barts Health NHS Trust includes two large tertiary referral centres (St Bartholomew's Hospital and Royal London Hospital), and two district general hospitals (Whipps Cross University Hospital and Newham University Hospital). We received approval from the Health Research Authority (19/HRA/0319) and Discovery Data Service board (dated September 24, 2019) for this study. All data were held on the Barts Health NHS Trust information technology network. We developed a statistical analysis plan before analysis (Supplementary material). No sample size calculation was required for this observational cohort study. We report our findings in line with the RECORD (Reporting of studies Conducted using Observational Routinely collected Data) statement.^14^

### Cohort identification

The local CCG areas included in primary care linkage are Tower Hamlets, Newham, City & Hackney, and Waltham Forest. Secondary care records were linked to local primary care records via the Discovery Data Service using an established pseudonymisation method. The Discovery Data Service holds complete data extracts from the primary care records system for patients in east London. Data extraction and linkage steps are outlined in the supplementary methods. We identified the first surgical procedure performed among patients registered at a relevant GP practice, aged ≥18 yr in any Barts Health NHS Trust hospital between January 1, 2012 and January 1, 2017 after a 5-yr washout period (January 1, 2007–December 31, 2011). We implemented the washout period to remove the prevalent pool effect.^15^ This negated the issue of a procedure we identified as being the first actually representing re-intervention after a procedure that occurred just before our selection window. Surgical procedures were identified using of Office of Population Censuses and Surveys Classification of Interventions and Procedures version 4 codes (OPCS codes).^16^ These are procedures typically performed in an operating theatre, under regional or general anaesthesia or involving insertion of a stent/device.^1^ We excluded obstetric surgical procedures and those involving organ donation.

### Outcome measures

The primary outcome measure was the number of healthcare contact days, defined as the number of days which involved contact with a healthcare professional expressed as number of days per patient year (Supplementary methods). Secondary outcome measures were healthcare contact days for individual types of contact (primary care, outpatient, inpatient, emergency department), days alive and at home within 90 days after surgery, and death within 2 yr of index surgery.^17^

### Healthcare encounters

To ensure we measured risk-time for each patient appropriately, we constructed windows of primary care registration. Registration windows started on the date of registration and ended on the earliest of the end of GP registration, the date of death or 731 days after surgery. Length of hospital stay after the index surgical procedure contributed to risk time and encounters, irrespective of GP registration. We identified all clinical encounters in primary care based on user role and consultation type. Each day with a clinical encounter was considered 1 day in contact. We identified all hospital admissions in the 2 yr before and after surgery. Each day spent in hospital was considered 1 day in contact. We identified attended outpatient appointments in the 2 yr before and after surgery based on attendance codes.^18^ Finally, we identified all emergency department attendances in the 2 yr before and after surgery. If patients had more than one encounter on a single day, this was counted as 1 day in contact. We did not use different weights for each type of encounter, but present them separately. We restricted all encounters to those that occurred during a primary care registration window.

### Derivation of covariates

We used age (in completed years) and sex (reported as male or female) recorded at the start of the index surgical episode in secondary care records. Ethnicity was aggregated into one of five categories (South Asian/Black/White/Other/Missing) from primary care records. Ethnic categories were based on the 18 categories of the UK 2011 census and were combined into four groups reflecting the study population: White (British, Irish, other White), Black (Black African, Black Caribbean, Black British, other Black, and mixed Black), South Asian (Bangladeshi, Pakistani, Indian, Sri Lankan, British Asian, other Asian, or mixed Asian), and Other (Chinese, Arab, any other ethnic group).^19^ Individuals of mixed ethnicity were grouped with their parent ethnic minority for the purposes of this study. The English Index of Multiple Deprivation (IMD) (2019) was used as a measure of social deprivation. The IMD score for each patient was mapped to the patient lower super output area of residence to derive national quintiles for the study population based on each patient's area of residence nearest to the time of index surgery.^20^ Admission category was based on admission method codes (elective or emergency).^18^ Procedures were grouped based on anatomical location of the index surgical procedure.^21^ Long-term diseases were captured from primary and secondary care records in the 2 yr before surgery. In secondary care, we used the International Classification of Diseases, 10th Revision (ICD-10) codes to capture Charlson Comorbidity Index diseases.^22^ We used a 2-yr look back file including inpatient and outpatient care to identify diagnoses associated with each patient before surgery. In primary care records, we used Read codes to capture Charlson Comorbidity Index diseases.^23^ In secondary care records, diseases were defined using a modified Charlson score.^24^ Presence of a relevant code in either primary or secondary care was sufficient for diagnosis of any disease. We identified the BMI and smoking status (never, former, current, or unknown) closest to the time before surgery in primary care data.

### Statistical analysis

We present separate analyses according to admission category (elective or emergency). We present all numbers of healthcare encounter per patient year with associated 95% confidence intervals calculated using Wald's exact methods. Because registration time and encounters are limited by the date of death, this partly accounts for the competing endpoint of death. We present the values before and after surgery, and the difference between these. We tested the change in rate using the rate ratio test.^23^ To demonstrate the characteristics associated with differing healthcare use after surgery, we stratified the cohort to quintiles of change of healthcare use and present the characteristics of each. We present the distribution of the change in healthcare use, and temporal changes in healthcare use by dividing the study period into eight 6-month blocks. We present the proportion patients days in contact with healthcare for sequential 7-day windows across the study period. We use a unit invariant knee method to identify potential inflection points in postoperative care trajectories.^25^ All analyses were performed using R (R Core Team, Vienna, Austria).

We defined clinically relevant patient populations according to 90-day risk of death to explore changes in burden of healthcare after surgery amongst surviving patients, and test the assumption that patients recover to pre-surgical levels of healthcare use regardless of this risk. We selected 90 days at the endpoint for risk modelling because the rate of death doubles by this point compared with 30 days, and in a recent study of emergency general surgery, the median survival time was 67 days.^1^^,^^26^ We developed logistic regression models for elective and emergency surgery separately. For both models, 90-day mortality was the outcome variable. Predictor variables were age (modelled with a restricted spline with three degrees of freedom), smoking status (never/former/current/unknown), presence of chronic diseases (entered individually), procedure group (one of 18 categories), inpatient or day-case status, index of multiple deprivation national quintile (categorical variable: 1/2/3/4/5). We applied this prediction model to each patient to generate a risk score. We present the characteristics, healthcare use and other outcomes for three risk groups ordered within the cohort from highest to lowest probability of death:

• High risk: containing 80% of all deaths.
• Moderate risk: containing 81–99% of deaths.
• Low risk: excluding 99% of all deaths (i.e. a low risk group accounting for 1% of deaths).

### Sensitivity analyses

The definition of surgery used includes a broad range of procedures which may be performed for reasons ranging from improved quality of life to the immediate preservation of life. We previously identified OPCS 4.7 that define procedures associated with substantial tissue damage to identify major procedures. To demonstrate how long-term healthcare use varies with the underlying surgical procedure, we stratified the cohort into major or non-major surgery using these codes. We present healthcare days and mortality associated with major and non-major procedures. The age of each patient increased over the 4-yr study period which may be associated with increased healthcare use. We calculated the proportion of patient days involving healthcare contact for each year of age excluding the 10 days before and after surgery and determined the rate of change as age increased by 4 yr. We modelled this using a linear regression model and entered age as a continuous variable.

### *Post hoc* analyses

We undertook a *post hoc* multivariable, negative binomial model to outline the association between baseline features and postoperative days in use with healthcare.

## Results

**Fig 1:**
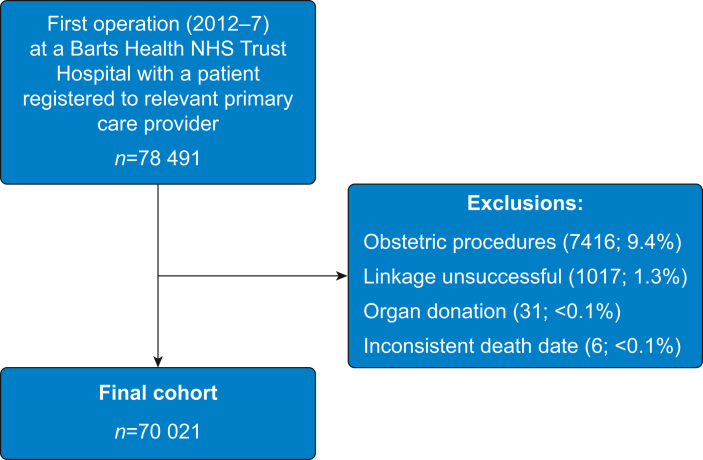
Summary of inclusion in study cohort.

We identified 78 491 first surgical procedures which met our inclusion criteria. We successfully linked to primary care records in 77 474 (98.7%) of identified patients. We excluded patients undergoing obstetric surgery (7416, 9.5%), organ donor surgery (31, <0.1%), and or an implausible date of death (6, <0.1%) (Fig 1).

### Characteristics of included patients

The analysis cohort included 70 021 patients. For 51 693 (73.8%) patients, the first surgical procedure was performed on an elective basis providing healthcare data describing 95 741 patient years before surgery and 96 172 patient years after surgery. For 18 328 (26.2%) patients, the first surgical procedure was performed as an emergency providing healthcare data describing 32 738 patient years before surgery and 30 990 patient years after surgery. Female upper genitourinary tract procedures were the most common category of surgery (6885; 9.8%). Ethnicity data were available for 64 734 (92.4%) patients, of whom 33 229 (47.6%) were White, 18 796 (26.8%) were South Asian, 9009 (12.9%) were Black, and 3700 (5.3%) were recorded as ‘Other’ ethnicity. Deprivation data were available for 69 893 (99.8%) patients, and 20 441 (29.1%) people resided in an area in the most deprived national quintile for social deprivation. At least one long-term disease was present in 34 919 (49.9%) patients, the most common being respiratory disease (14 469; 20.7%). At the time of surgery, 16 332 (23.3%) patients were smokers (Table 1). Older patients, those of South Asian ethnicity and those with a higher burden of long-term disease more frequently experienced an increase in healthcare use after surgery (Supplementary results).

**Table 1:** Characteristics of included patients, presented by admission category. Data are presented as number (%) unless otherwise stated. Admission category is based on admission method codes. Index of multiple deprivation quintile based on patient residential lower super-output area. sd, standard deviation; IQR, inter-quartile range.

	All	Admission category	
		Elective	Emergency
Number	70 021	51 693 (73.8%)	18 328 (26.2%)
Age, yr			
Mean (sd)	49.8 (19)	49.2 (18.2)	51.4 (20.8)
Median (IQR)	49 (33–65)	48 (34–63)	50 (33–69)
Sex			
Male	33 091 (47.3%)	22 811 (44.1%)	10 280 (56.1%)
Female	36 930 (52.7%)	28 882 (55.9%)	8048 (43.9%)
Index of multiple deprivation			
Most deprived 1	20 411 (29.1%)	14 729 (28.5%)	5682 (31%)
2	35 716 (51%)	26 459 (51.2%)	9257 (50.5%)
3	9566 (13.7%)	7281 (14.1%)	2285 (12.5%)
4	3153 (4.5%)	2349 (4.5%)	804 (4.4%)
Least deprived – 5	1047 (1.5%)	783 (1.5%)	264 (1.4%)
Missing	128 (0.2%)	92 (0.2%)	36 (0.2%)
Ethnic group			
White	33 229 (47.5%)	24 429 (47.3%)	8800 (48%)
South Asian	18 796 (26.8%)	13 880 (26.9%)	4916 (26.8%)
Black	9009 (12.9%)	6833 (13.2%)	2176 (11.9%)
Other	3700 (5.3%)	2772 (5.4%)	928 (5.1%)
Missing	5287 (7.6%)	3779 (7.3%)	1508 (8.2%)
Smoking status			
Never	32 701 (46.7%)	24 950 (48.3%)	7751 (42.3%)
Former	17 803 (25.4%)	13 146 (25.4%)	4657 (25.4%)
Current	16 332 (23.3%)	11 389 (22%)	4943 (27%)
Missing	3185 (4.5%)	2208 (4.3%)	977 (5.3%)
Number of long-term diseases			
0	35 102 (50.1%)	27 389 (53%)	7713 (42.1%)
1	19 621 (28%)	14 803 (28.6%)	4818 (26.3%)
2	8777 (12.5%)	5976 (11.6%)	2801 (15.3%)
≥ 3	6521 (9.3%)	3525 (6.8%)	2996 (16.3%)
BMI, kg m^−2^			
Mean (sd)	26.9 (5.9)	26.9 (5.9)	26.6 (5.9)
Median (IQR)	26 (22.8–30)	26.2 (22.9–30.1)	25.8 (22.5–29.7)
Missing	6230 (8.9%)	4186 (8.1%)	2044 (11.2%)

### Patient outcomes after elective surgery

**Fig 2:**
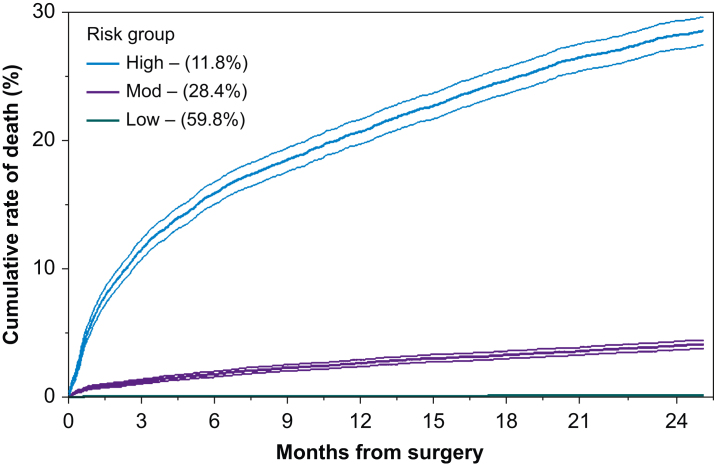
Cumulative rate of death, stratified by risk group among patients undergoing elective surgery. Shaded area indicates the 95% confidence interval; numbers in brackets are the proportion of patients undergoing elective surgery within each risk group.

**Fig 3:**
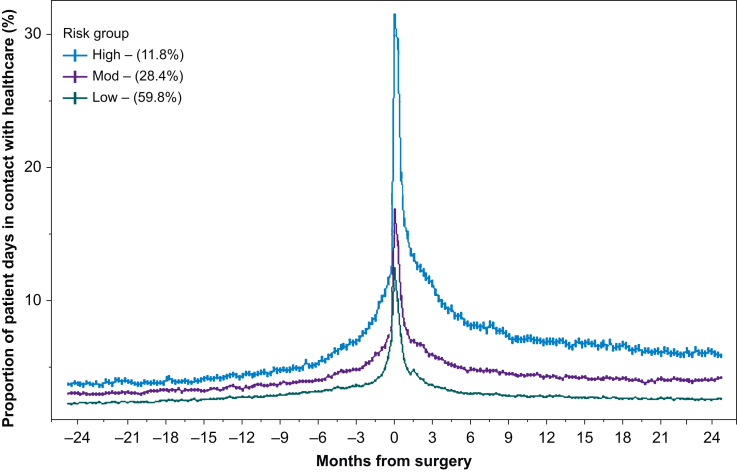
Proportion of patient days in contact with healthcare in the 2 yr before and after surgery, stratified by risk group among patients undergoing elective surgery. Data aggregated by 7-day windows, each of which has 95% confidence intervals. Each point represents the proportion of patient days spent in contact with healthcare during the 7-day window. All patients contact healthcare on Day 0 (i.e. the date of surgery), and this was excluded from modelling to prevent distortion. Numbers in brackets are the proportion of patients undergoing elective surgery within each risk group.

We included 51 693 patients undergoing elective surgery, of whom 214 (0.4%) died within 90 days and 1867 (3.6%) died within 2 yr (Supplementary Fig. S1). The median number of days-alive at home within 90 days was 88 days (inter-quartile range [IQR], 86–90 days). For the 2 yr elective surgery, 12.7 (12.7–12.7) days per patient year involved contact with a healthcare professional, increasing to 15.5 days (15.4–15.5 days) per patient year (rate ratio=1.22 [1.21–1.22]) (Table 2). The greatest number of days in contact with healthcare was in the 6 months immediately after surgery (21.9 days per patient year) (Supplementary Table S2). Within the elective surgery population, 6071 (11.7%) patients were categorised as high-risk, 14 698 (28.4%) as moderate risk and 30 924 (59.8%) as low-risk (Supplementary Table S3). The number of healthcare contact days increased after surgery for all three risk groups, but this increase was greatest and persisted the longest among high-risk patients (Fig 2, Fig 3, Supplementary Fig. S3).

**Table 2:** Number of days in contact before and after surgery, stratified by type of encounter and presented by admission category risk groups. Data presented as encounters per patient year with associated 95% confidence intervals, unless otherwise stated. Before, 2 yr before surgery; After, 2 yr after surgery. ∗*P*<0.05. ^**†**^Excludes the days spent in hospital for index surgical admission.

Timing	Primary care encounters			Secondary care encounters									Total healthcare encounters		
				Outpatient attendance			Emergency department			Hospital admissions					
	Before	After	Before		After	Before		After	Before		After†	Before		After	Rate ratio
All	9.6 (9.5–9.6)	9.4 (9.4–9.5)	2.2 (2.2–2.2)		3.9 (3.9–3.9)	0.3 (0.3–0.4)		0.5 (0.5–0.5)	1.3 (1.3–1.3)		3.0 (3.0–3.0)	13.0 (13.0–13.0)		17.7 (17.7–17.8)	1.37 (1.36–1.37)∗
*Elective surgery*															
All	9.6 (9.5–9.6)	9 (9–9)	2.5 (2.4–2.5)		3.9 (3.8–3.9)	0.3 (0.3–0.3)		0.4 (0.4–0.4)	0.7 (0.7–0.7)		2.2 (2.1–2.1)	12.7 (12.7–12.7)		15.5 (15.4–15.5)	1.22 (1.21–1.22)∗
Low risk	8.2 (8.2–8.2)	7.4 (7.4–7.4)	2.2 (2.2–2.2)		3 (3–3)	0.3 (0.3–0.3)		0.4 (0.3–0.4)	0.4 (0.4–0.4)		0.9 (0.9–0.9)	10.8 (10.8–10.9)		11.6 (11.6–11.6)	1.07 (1.07–1.07)∗
Moderate risk	10.9 (10.8–10.9)	10.5 (10.5–10.6)	2.5 (2.5–2.5)		4.3 (4.3–4.4)	0.3 (0.3–0.3)		0.4 (0.4–0.5)	0.8 (0.8–0.8)		2.6 (2.6–2.6)	14.1 (14–14.1)		17.9 (17.8–17.9)	1.27 (1.27–1.28)∗
High risk	13 (13–13.1)	14 (13.9–14)	3.5 (3.5–3.6)		7.4 (7.3–7.4)	0.3 (0.3–0.3)		0.7 (0.7–0.7)	2.2 (2.2–2.2)		7.7 (7.7–7.8)	18.5 (18.4–18.5)		30.4 (30.3–30.5)	1.64 (1.63–1.65)∗
*Emergency surgery*															
All	9.5 (9.5–9.5)	10.7 (10.7–10.7)	1.3 (1.3–1.3)		3.9 (3.8–3.9)	0.5 (0.5–0.5)		0.8 (0.8–0.8)	2.9 (2.9–2.9)		5.6 (5.6–5.7)	13.8 (13.7–13.8)		24.8 (24.8–24.9)	1.8 (1.80–1.81)∗
Low risk	6.2 (6.2–6.3)	7.4 (7.3–7.4)	1 (1–1)		2.9 (2.8–2.9)	0.5 (0.5–0.5)		0.6 (0.6–0.6)	1 (1–1)		1.5 (1.5–1.6)	8.4 (8.3–8.4)		13.3 (13.2–13.4)	1.59 (1.57–1.6)∗
Moderate risk	9.6 (9.5–9.6)	11.5 (11.5–11.6)	1.1 (1.1–1.1)		4 (3.9–4)	0.4 (0.4–0.4)		0.7 (0.7–0.7)	2.4 (2.4–2.4)		5 (4.9–5)	13.2 (13.1–13.2)		24.8 (24.7–24.9)	1.88 (1.87–1.89)∗
High risk	13.7 (13.6–13.8)	16.1 (16–16.2)	2 (2–2)		5.7 (5.7–5.8)	0.6 (0.6–0.6)		1.3 (1.3–1.3)	6.1 (6.1–6.2)		15.8 (15.7–15.9)	21.6 (21.5–21.7)		49.2 (49–49.4)	2.28 (2.26–2.29)∗

### Patient outcomes after emergency surgery

We included 18 328 patients undergoing emergency surgery, of whom 1024 (5.5%) died within 90 days and 1867 (14.3%) died within 2 yr (Supplementary Fig. S1). The median number of days-alive at home within 90 days was 84 (IQR, 74–88) days. For the 2 yr before emergency surgery, 13.8 (13.7–13.8) days per patient year involved contact with a healthcare professional increasing to 24.8 (24.8–24.9) days after surgery (rate ratio, 1.8 [1.8–1.8]) (Table 2 and Fig 3). The greatest number of days in contact with healthcare was in the 6 months immediately after surgery (46.6 days per patient year) (Supplementary Table S2). Within the emergency surgery population, 4459 (24.3%) patients were categorised as high risk, 7248 (39.5%) as moderate risk group, and 6621 (36.1%) as low risk (Supplementary Table S3). The number of healthcare contact days increased even more markedly after emergency surgery, and this increase was once again greatest for high-risk patients (Supplementary Figs 2 and 3). A summary of each model is in the Supplementary results and Supplementary Table 4.

### Changes in different types of healthcare contact

Most healthcare encounters were with primary care both before (elective, 9.6 days; emergency, 9.5) and after (elective, 9.0 days; emergency, 10.7) surgery. The largest change in encounter types was inpatient admissions after elective (rate ratio, 2.2 [2.1–2.2]), and emergency (rate ratio: 3.66 [3.63–3.68]) surgery. High-risk patients experienced the greatest increase in healthcare use after surgery across all types of encounters, despite their higher baseline of healthcare use. In the 2 yr after surgery, high-risk patients experienced a more than doubling in all secondary care encounter types. Table 2 summarises the change in type of healthcare contacts in the 2 yr before and after surgery, stratified for each emergency and elective risk group.

### Sensitivity analyses

We identified 19 236 patients (27.5%) undergoing major surgery, the increase in healthcare use was greatest among patients undergoing major surgery (rate ratio, 1.61 [1.60–1.61]) (Supplementary Table S5). Across the 4-yr study period, the rate of increase in healthcare use ranged from 0.2 to 0.8 encounters (Supplementary results and Supplementary Fig. S4).

### *Post hoc* analyses

We generated a negative-binomial model to outline features associated with postoperative healthcare use (Supplementary Table S6). The features most strongly associated with elevated postoperative healthcare use were higher baseline healthcare use, advancing age and a diagnosis of cancer.

## Discussion

The principal finding of this study is that patients at high risk of death after surgery experience large and persistent increase in healthcare utilisation in the 2 yr after surgery, even if they survive the immediate perioperative period. Most increase in healthcare use occurred in the first 6 months after surgery and a new, higher baseline is typically established around 1 yr after surgery. Increased age, long-term disease, and emergency surgical procedures are associated with more healthcare use at all time points. Although the greatest relative increase in healthcare use is in secondary care settings, most healthcare contacts were in primary care both before and after surgery.

Most prior studies of surgical outcomes focus on immediate complications and death after surgery, but few have described how the trajectory of healthcare use changes in the years after surgery. We compared data before and after surgery, including information from primary and secondary care. Most patients have a modest increase in healthcare use after surgery, but for certain patient groups the need for surgery reflects a transition point towards a much higher healthcare use. Trends in healthcare contact increase in the 6 months before surgery and peak in the 6 months after surgery. This broadly aligns with prior reports have suggested that unplanned readmissions persist throughout the first 3 months after surgery.^27^ A new, higher baseline of healthcare use is established around 1 yr after surgery. This new baseline would suggest that the increase in healthcare use resolves around 1 yr after surgery among survivors, and that this period is a suitable window to capture postoperative harms associated with surgery. Emergency surgery is associated with greater healthcare use immediately before and after surgery, and a greater healthcare need after surgery among survivors. Our findings add to the body of evidence regarding the poor short- and long-term outcomes experienced by high-risk surgical patients.^5^, ^6^, ^7^^,^^21^^,^^22^ An important difference between our study and others of the high-risk population is the definition of high-risk patients. Most prior studies used a cut-off, based on the risk of death associated with procedures.^5^^,^^28^ We created a simple risk model and used this to estimate risk for everyone in the dataset. We identified patients accounting for 80% of all deaths after surgery; these accounted for 11.7% of elective procedures and 24.3% of emergency procedures. This definition is highly reproducible and transferable, with the exact cut-off considered high-risk varying depending on the setting, while still accounting for the distribution of deaths. This can be repeatedly updated to account for temporal improvements in outcomes. In both elective and emergency settings, high-risk patients were older with a higher burden of long-term disease, with twice as many days in contact with healthcare before surgery as low-risk surgical patients. Our findings are consistent with previous reports of associations between higher preoperative healthcare use and poor patient outcomes.^24,^^32^, ^33^ By 2 yr after surgery, low-risk patients had one further day in contact with healthcare, whereas high-risk patients had an increase of 10 days. High-risk patients have greater healthcare use before surgery but experience a disproportionate increase which persists after the immediate postoperative period. These findings have important implications for shared decision-making. Although there is much focus on the risk of early death after surgery, our findings would suggest that there are persistent changes in healthcare use among those who survive the immediate postoperative period. For high-risk surgical patients, this may represent a substantial increase in time spent with healthcare. In addition to this, there are likely wider costs to patients associated with increased healthcare encounters, such as need for transport, reduced opportunity for employment, and less time to spend with family or friends. More broadly, we anticipate that patients with higher healthcare use likely have lower quality of life, although this was beyond the scope of the present study.^29^

The surgical population is ageing more rapidly than the general population; by 2030 a further 500 000 patients aged more than 75 yr will have surgery.^2^ Advanced age is a key feature of high-risk patients, so it is likely that as more older patients undergo surgery, the size of the high-risk population will increase.^5^ This is important for policymakers as they plan future primary and secondary care services to accommodate to this need. Although we identified that the largest increase in healthcare contacts was in secondary care, most healthcare was provided by primary care both before and after surgery. Although serious complications, such as anastomotic leak, may require an unplanned hospital re-admission, we anticipate that a substantial portion of postoperative complications are managed within primary care. Prior studies have highlighted the potential benefits of early primary care review after surgery.^34^^,^ These interventions may be best targeted at high-risk patients.^31^ In the context of mounting pressures on primary and secondary care, the effectiveness of this approach to improve outcomes is unclear.^,^ However, the very large size of the surgical population means that even modest reductions in postoperative healthcare use will likely have a meaningful impact on the wider healthcare system. Although we measured days in contact with healthcare, the impact on a patient of a hospital admission is likely greater than a primary care attendance. Instead of providing an arbitrary weighting to each encounter, we present the days in contact, stratified by type of encounter for readers to consider. Additional research is required to determine what is leading to the increased healthcare need among high-risk patients. This will enable development of appropriate prevention strategies. These strategies are likely to include preoperative optimisation, perioperative treatments to reduce complications and postoperative interventions to enhance recovery.^30^^,^^34^, ^35^, ^36^, ^37^ Identification of high-risk patients before their operation, and a detailed understanding of how interventions may affect subsequent healthcare need, will facilitate development of these care pathways.

This analysis has strengths and weaknesses. We used an established methodology to link detailed primary and secondary care records. Our linkage rate was more than 98%, indicating this was highly successful. We handled differential exposure time before and after surgery by using a time at risk calculation. We used a reproducible definition of surgery based on OPCS codes, enabling comparison with other studies. Finally, we obtained robust death data from the NHS Spine via an established route. This analysis also has limitations. The external validity of our findings are limited by the high rate of deprivation in our cohort (more than 80% come from the two most deprived quintiles nationally), high prevalence of long-term diseases (50% having at least one disease), and the high rate of emergency surgery which accounted for one-quarter of procedures. The increased prevalence of chronic diseases compared with national data may partly reflect better recording in primary care records or the high burden of disease experienced by the East London population. Our definition of surgery is broad as we aim to describe the totality of surgical activity, so we did a sensitivity analysis, focusing on major surgery. Patients undergoing major and non-major surgery had very similar healthcare use before surgery, but those requiring major surgery had greater healthcare use after surgery. Some patients may have been admitted to hospitals away from the four we captured data from, but we anticipate this would not be differential by high-risk surgery status and therefore not bias our findings. Handling surgical procedure codes within risk modelling is challenging given the very large number of possible operations, and we took a pragmatic approach to collapse these into broad anatomical categories. Our risk model was intentionally simple and should not be used for patient-level risk estimation.

## Conclusions

Patients at high-risk of early death after surgery experience large and persistent increase in healthcare burden in the 2 yr after surgery. For these patients, surgery reflects an inflection point towards higher healthcare utilisation. Further research should explore the reasons why high-risk patients experience higher healthcare use to inform development of strategies to reduce this healthcare need. Information regarding expected changes in healthcare use after surgery may aid patients as they decide on the most appropriate treatment.

## Authors' contributions

Study design: AJF, RP, JP

Data collection and analysis: AF, KB

Data interpretation: all authors

Writing the first draft of the manuscript: AF, JP.

All authors revised the manuscript for important intellectual content and approved the final version.

Access to the data and act as guarantors: AJF, JP

## Declarations of interest

AJF holds a National Institute for Health Research Doctoral Research fellowship (DRF-2018-11-ST2-062). RP has received honoraria and/or research grants from 10.13039/100006520Edwards Lifesciences, Intersurgical and 10.13039/100004330GlaxoSmithKline within the past 5 yr and holds editorial roles with the *British Journal of Anaesthesia* and the *British Journal of Surgery*. KB and JP report no relevant conflicts of interest.

## Funding

NIHR Doctoral Research Fellowship (DRF-2018-11-ST2-062 to AJF). The funding source had no role in the study design, data collection, analysis, interpretation, or writing the report.

## Data availability

This study used patient level data at Barts Health NHS Trust linked to primary care records. It is not possible to share raw patient-level data without approvals from the Health Research Authority and the data controllers.
