# Supplementary material for: Change in healthcare utilisation after surgical treatment: observational study of routinely collected patient data from primary and secondary care
Source: Br J Anaesth. 2022 Oct 1;129(6):889–97. doi: 10.1016/j.bja.2022.07.012 (PMC9748995; doi:10.1016/j.bja.2022.07.012)
Supplement: Multimedia component 1 [file mmc1.docx]

**Change in burden of healthcare after surgical treatment:**

**Observational study of routinely collected patient data from primary and secondary care.**

**SUPPLEMENTARY FILE**

**Supplementary methods**

*Data extraction*

From Barts Health we extracted data on in-hospital stay from the Cerner Millennium warehouse for the two years before and after the index surgical procedure. This included episode information (dates of admission and discharge, type of admission, location of treatment), diagnostic information (coded using the international statistical classification of diseases and related health problems, 10^th^ revision [ICD-10]) and procedures (coded using OPCS). We extracted date, type and attendance status for all outpatient appointments. We extracted date of all emergency department attendances. From the Discovery Data Service we extracted data for relevant patients in the two years before and after surgery. This included dates of GP registration, all primary care encounters, diagnostic information (coded with Read codes) and specified medications (identified by BNF Chapter 4.1 for antibiotic prescription). The Discovery Data Service also provided date of death from the NHS Spine.

*Linkage*

Linkage was undertaken by a trusted third party using existing infrastructure. NHS Numbers were pseudonymised using the Open Pseudonymiser platform, and these salted NHS numbers were used to select relevant records within Discovery. Data were extracted by analysts at the Discovery Data Service, and was transferred using a secure file transfer protocol by the information technology team at Barts Health. A unique study identifier, common to both datasets, was used to link primary and secondary care data at patient level.

*Calculation of days per patient year in contact with a healthcare professional*

$$events=\sum Number of days in contact with a healthcare professional during risk time$$

$$risk time=\sum Number of days registered with a relevant primary care provider$$

$$\left\{ \frac{events}{risk time} \right\} \times365.25=Days per patient year in contact with a healthcare professional$$

**Supplementary results**

The three most influential variables in each risk model, as assessed by Chi^2^ statistics, were age (elective: 76.4, p = 0.000; emergency: 199.6, p<0.01), procedure group (elective: 80.4, p=0.000; emergency: 185.7, p<0.01) and the presence of cancer (elective: 76.5, p=0.000; emergency: 226.5, p<0.01). The overall C-statistic in the emergency model was 0.87 (95%CI: 0.86 to 0.88), the high-risk group were identified by a cut-off of 6.8% estimated risk of 90-day death above which the positive predictive value was 0.18 and the negative predictive value 0.985. The overall C-statistic in the elective model was 0.93 (95%CI: 0.92 to 0.94), the high-risk group were identified by a cut-off of 0.69% estimated risk of 90-day death above which the positive predictive value was 0.03 and the negative predictive value was 0.99.

*Influence of increasing age on healthcare use*

All patients who survived the entirety of the study period aged by four years. We therefore determined the influence of a four-year increase in age on healthcare use. Across a four-year difference in age, the increase in healthcare use ranged from 0.2 days per patient year among patients aged 20-24 years, to 0.8 days among patients aged 80-84 years (supplementary figure X).

*Characteristics associated with higher healthcare use after surgery*

When we stratified elective patients to quintiles of change in healthcare use, 30,522 (59.0%) had an increase in healthcare use (quintiles 3-5). Compared to those in the lowest quintile, patients in the highest quintile were older (mean: 57.4±18 years vs. 46.0±17.7 years), had more chronic diseases (≥3 diseases: 25.4% vs. 6.4%), and were more frequently of South Asian ethnicity (46.0% vs. 27.4%) (supplementary table 1). When we stratified emergency patients into quintiles of change in healthcare use, 14,416 (78.7%) had an increase in healthcare use (quintiles 2-5), the features associated with increased healthcare use in emergency patients were the same as elective patients.

|  | **Quintile of change** | | | | | | | | | | | | | | | | |
| --- | --- | --- | --- | --- | --- | --- | --- | --- | --- | --- | --- | --- | --- | --- | --- | --- | --- |
|  | **1** | | | | **2** | | **3** | | | | **4** | | | | **5** | | |
| **Median (IQR) days change** | -8 (-12.5 to -6) | | | | -2.5 (-3.5 to -1.5) | | 0.5 (0 to 1.5) | | | | 4.6 (3.5 to 6.5) | | | | 16 (11.5 to 28.5) | | |
| **Number** | 10175 | | | | 10174 | | 10174 | | | | 10174 | | | | 10174 | | |
| *Age* | | | | | | | | | | | | | | | | | |
| **Mean (SD)** | 46 (17.7) | | | | | 45.3 (17) | 47.1 (17.6) | | | | 50.7 (18.2) | | | | 57.4 (18) | | |
| *Sex* | | | | | | | | | | | | | | | | | |
| **Male** | 4076 (40.1%) | | | | | 5403 (53.1%) | 4706 (46.3%) | | | | 4489 (44.1%) | | | | 4348 (42.7%) | | |
| **Female** | 6099 (59.9%) | | | | | 4771 (46.9%) | 5468 (53.7%) | | | | 5685 (55.9%) | | | | 5826 (57.3%) | | |
| *Day-case or inpatient* | | | | | | | | | | | | | | | | | |
| **Day-case** | | | | 2129 (20.9%) | 8319 (81.8%) | | | | 8002 (78.7%) | | | | 2988 (29.4%) | | | 5592 (55%) | |
| **Inpatient** | | | | 8046 (79.1%) | 1855 (18.2%) | | | | 2172 (21.3%) | | | | 7186 (70.6%) | | | 4582 (45%) | |
| *Index of multiple deprivation (quintile)* | | | | | | | | | | | | | | | | | |
| **Most deprived - 1** | | | | 2987 (29.4%) | 2832 (27.8%) | | | | 1457 (14.3%) | | | | 1366 (13.4%) | | | 1442 (14.2%) | |
| **2** | | | | 5116 (50.3%) | 5313 (52.2%) | | | | 2816 (27.7%) | | | | 5235 (51.5%) | | | 2962 (29.1%) | |
| **3** | | | | 1448 (14.2%) | 155 (1.5%) | | | | 5297 (52.1%) | | | | 2928 (28.8%) | | | 5052 (49.7%) | |
| **4** | | | | 454 (4.5%) | 1441 (14.2%) | | | | 125 (1.2%) | | | | 472 (4.6%) | | | 516 (5.1%) | |
| **Least deprived - 5** | | | | 155 (1.5%) | 415 (4.1%) | | | | 463 (4.6%) | | | | 158 (1.6%) | | | 182 (1.8%) | |
| **Missing** | | | | 15 (0.1%) | 18 (0.2%) | | | | 16 (0.2%) | | | | 15 (0.1%) | | | 20 (0.2%) | |
| *Ethnic group* | | | | | | | | | | | | | | | | | |
| **White** | | 4841 (47.6%) | | | 4693 (46.1%) | | | 4612 (45.3%) | | | | 2896 (28.5%) | | | | 765 (7.5%) | |
| **South Asian** | | 2793 (27.4%) | | | 1376 (13.5%) | | | 1402 (13.8%) | | | | 4675 (46%) | | | | 5235 (51.5%) | |
| **Black** | | 1227 (12.1%) | | | 2772 (27.2%) | | | 2811 (27.6%) | | | | 1385 (13.6%) | | | | 2409 (23.7%) | |
| **Other** | | 623 (6.1%) | | | 700 (6.9%) | | | 540 (5.3%) | | | | 480 (4.7%) | | | | 1317 (12.9%) | |
| **Missing** | | 691 (6.8%) | | | 633 (6.2%) | | | 809 (8%) | | | | 738 (7.3%) | | | | 448 (4.4%) | |
| *Number of chronic diseases* | | | | | | | | | | | | | | | | | |
| **0** | | | 5604 (55.1%) | | 2584 (25.4%) | | | | | 2717 (26.7%) | | | | 5394 (53%) | | | 3196 (31.4%) |
| **1** | | | 2811 (27.6%) | | 6490 (63.8%) | | | | | 6196 (60.9%) | | | | 1174 (11.5%) | | | 3497 (34.4%) |
| **2** | | | 1111 (10.9%) | | 730 (7.2%) | | | | | 861 (8.5%) | | | | 2976 (29.3%) | | | 892 (8.8%) |
| **≥3** | | | 649 (6.4%) | | 370 (3.6%) | | | | | 400 (3.9%) | | | | 630 (6.2%) | | | 2589 (25.4%) |

**Supplementary table 1. Characteristics of patients, stratified by quintile of healthcare use change after elective surgery.** Data are presented as number (%) unless otherwise stated. SD is standard deviation; IQR is inter-quartile range. Admission category is based on admission method codes. Index of multiple deprivation quintile based on patient residential lower super-output area.

|  | **Days from surgery** | | | | | | | |
| --- | --- | --- | --- | --- | --- | --- | --- | --- |
|  | **-730 to -548** | **-547 to -365** | **-364 to -182** | **-181 to 0** | **1 to 181** | **182 to 364** | **365 to 547** | **548 to 730** |
| *Elective surgery* | | | | | | | | |
| **Days in contact with healthcare**  **(per patient year)** | 10.1  (10.1 to 10.1) | 10.9  (10.8 to 10.9) | 12.3  (12.3 to 12.4) | 17.3  (17.2 to 17.3) | 21.9  (21.8 to 21.9) | 14.2  (14.1 to 14.2) | 13.1  (13 to 13.1) | 12.5  (12.5 to 12.6) |
| **Cumulative rate of death (%)** | - | - | - | - | 0.9  (0.8 to 1) | 2  (1.8 to 2.1) | 2.9  (2.7 to 3) | 3.8  (3.6 to 3.9) |
| *Emergency surgery* | | | | | | | | |
| **Days in contact with healthcare**  **(per patient year)** | 10.4  (10.4 to 10.5) | 10.9  (10.8 to 10.9) | 11.8  (11.7 to 11.9) | 21.5  (21.4 to 21.6) | 46.6  (46.4 to 46.7) | 18.8  (18.7 to 18.9) | 16.4  (16.3 to 16.5) | 15.1  (15 to 15.2) |
| **Cumulative rate of death (%)** | - | - | - | - | 7.9  (7.5 to 8.3) | 10.9  (10.4 to 11.4) | 13.2  (12.7 to 13.7) | 15  (14.5 to 15.6) |

**Supplementary table 2. Number of days in contact with healthcare and cumulative rate of death, presented for eight six-monthly windows for two years before and after surgery, stratified by admission category.** Data are presented with 95% confidence intervals.

|  | **Elective surgery** | | | **Emergency surgery** | | |
| --- | --- | --- | --- | --- | --- | --- |
|  | Low risk | Moderate risk | High risk | Low risk | Moderate risk | High risk |
| **N** | 30924 (59.8) | 14698 (28.4) | 6071 (11.7) | 6621 (36.1) | 7248 (39.5) | 4459 (24.3) |
| *Age* | | | | | | |
| **Mean (SD)** | 39.2 (13.9) | 61.4 (12.8) | 70.7 (10.9) | 31 (9) | 56.5 (14.9) | 73.4 (12.5) |
| *Sex* | | | | | | |
| **Female** | 18304 (59.2%) | 7704 (52.4%) | 2874 (47.3%) | 3278 (49.5%) | 2707 (37.3%) | 2063 (46.3%) |
| **Male** | 12620 (40.8%) | 6994 (47.6%) | 3197 (52.7%) | 3343 (50.5%) | 4541 (62.7%) | 2396 (53.7%) |
| *Ethnic group* | | | | | | |
| **White** | 13779 (44.6%) | 7137 (48.6%) | 3513 (57.9%) | 2616 (39.5%) | 3579 (49.4%) | 2605 (58.4%) |
| **South Asian** | 8975 (29%) | 3786 (25.8%) | 1119 (18.4%) | 2157 (32.6%) | 1908 (26.3%) | 851 (19.1%) |
| **Black** | 4121 (13.3%) | 2043 (13.9%) | 669 (11%) | 804 (12.1%) | 920 (12.7%) | 452 (10.1%) |
| **Other** | 1793 (5.8%) | 711 (4.8%) | 268 (4.4%) | 444 (6.7%) | 329 (4.5%) | 155 (3.5%) |
| **Missing** | 2256 (7.3%) | 1021 (6.9%) | 502 (8.3%) | 600 (9.1%) | 512 (7.1%) | 396 (8.9%) |
| *Index of multiple deprivation (quintiles)* | | | | | | |
| **Most deprived - 1** | 8712 (28.2%) | 4091 (27.8%) | 1926 (31.7%) | 2010 (30.4%) | 2142 (29.6%) | 1530 (34.3%) |
| **2** | 15739 (50.9%) | 7669 (52.2%) | 3051 (50.3%) | 3340 (50.4%) | 3726 (51.4%) | 2191 (49.1%) |
| **3** | 4581 (14.8%) | 1964 (13.4%) | 736 (12.1%) | 885 (13.4%) | 923 (12.7%) | 477 (10.7%) |
| **4** | 1442 (4.7%) | 651 (4.4%) | 256 (4.2%) | 283 (4.3%) | 327 (4.5%) | 194 (4.4%) |
| **Least deprived - 5** | 450 (1.5%) | 231 (1.6%) | 102 (1.7%) | 103 (1.6%) | 94 (1.3%) | 67 (1.5%) |
| *Smoking status nearest surgery* | | | | | | |
| **Current** | 6826 (22.1%) | 3108 (21.1%) | 1455 (24%) | 1767 (26.7%) | 2112 (29.1%) | 1064 (23.9%) |
| **Former** | 5964 (19.3%) | 4675 (31.8%) | 2507 (41.3%) | 1043 (15.8%) | 2032 (28%) | 1582 (35.5%) |
| **Never** | 16747 (54.2%) | 6293 (42.8%) | 1910 (31.5%) | 3367 (50.9%) | 2733 (37.7%) | 1651 (37%) |
| **Unknown** | 1387 (4.5%) | 622 (4.2%) | 199 (3.3%) | 444 (6.7%) | 371 (5.1%) | 162 (3.6%) |
| *Number of chronic diseases* | | | | | | |
| **0** | 21946 (71%) | 5210 (35.4%) | 233 (3.8%) | 5067 (76.5%) | 2530 (34.9%) | 116 (2.6%) |
| **1** | 7334 (23.7%) | 5632 (38.3%) | 1837 (30.3%) | 1354 (20.5%) | 2582 (35.6%) | 882 (19.8%) |
| **2** | 1343 (4.3%) | 2692 (18.3%) | 1941 (32%) | 169 (2.6%) | 1402 (19.3%) | 1230 (27.6%) |
| **≥3** | 301 (1.0%) | 1164 (7.9%) | 2060 (33.9%) | 31 (0.5%) | 734 (10.1%) | 2231 (50.0%) |
| *90 days of surgery* | | | | | | |
| **Rate of death** | 0 (0 to 0) | 0.3 (0.2 to 0.3) | 2.8 (2.4 to 3.3) | 0.1 (0 to 0.2) | 2.7 (2.3 to 3.1) | 18.4 (17.3 to 19.5) |
| **DAY-90 (days)** | 89 (87 to 90) | 88 (85 to 89) | 84 (77 to 88) | 88 (85 to 89) | 84 (75 to 87) | 69 (22 to 82) |

**Supplementary table 3. Characteristics and rate of death within 90 days of surgery, stratified to risk groups based on a logistic regression model for elective and emergency groups separately.** Data are presented as number (%) unless otherwise stated. SD is standard deviation; IQR is inter-quartile range; 95% CI is 95% confidence interval; of multiple deprivation quintile based on patient residential lower super-output area. Rate of death is presented as percentage with 95% confidence interval. DAH-90; Days alive at home within 90-days reported as median with interquartile range.

| **Variable** | **Elective** | **Emergency** |
| --- | --- | --- |
| Age (Q1 Vs Q3) | 9.69 (3.95 to 23.76) | 6.13 (4.48 to 8.4) |
| Sex (Male) | 0.84 (0.62 to 1.14) | 0.88 (0.76 to 1.03) |
| Day-case (vs. inpatient) | 0.70 (0.50 to 0.95) | 0.46 (0.33 to 0.60) |
| **Charlson comorbidity index domains** | | |
| Cardiac failure | 2.36 (1.56 to 3.58) | 2.18 (1.83 to 2.6) |
| Myocardial infarction | 0.83 (0.51 to 1.36) | 0.88 (0.71 to 1.09) |
| Peripheral vascular disease | 1.41 (0.88 to 2.27) | 1.23 (0.98 to 1.54) |
| Stroke | 2.01 (1.36 to 2.97) | 1.44 (1.21 to 1.72) |
| Dementia | 1.03 (0.49 to 2.19) | 2.06 (1.65 to 2.57) |
| Respiratory disease | 1.63 (1.21 to 2.18) | 1.13 (0.97 to 1.32) |
| Rheumatological disease | 1.21 (0.62 to 2.37) | 0.97 (0.68 to 1.38) |
| Liver disease | 2.23 (1.23 to 4.04) | 2 (1.53 to 2.62) |
| Diabetes mellitus | 0.95 (0.69 to 1.32) | 1.02 (0.87 to 1.19) |
| Hemiplegia | 0.7 (0.16 to 3.09) | 0.76 (0.49 to 1.2) |
| Chronic kidney Disease | 0.86 (0.6 to 1.24) | 1.14 (0.96 to 1.34) |
| Cancer | 4.89 (3.43 to 6.98) | 3.61 (3.05 to 4.26) |
| Human Immuno-deficiency Virus | 2.77 (0.6 to 12.71) | 2.07 (1.03 to 4.17) |
| **Index of multiple deprivation (vs. 1; most deprived)** | | |
| 2 | 0.9 (0.59 to 1.36) | 0.98 (0.8 to 1.21) |
| 3 | 0.85 (0.55 to 1.32) | 0.99 (0.8 to 1.23) |
| 4 | 0.97 (0.63 to 1.49) | 1 (0.8 to 1.25) |
| 5 (least deprived) | 0.72 (0.46 to 1.13) | 0.91 (0.73 to 1.14) |
| **Ethnicity (vs. white)** | | |
| Asian | 1.24 (0.82 to 1.86) | 1.02 (0.84 to 1.24) |
| Black | 1.08 (0.67 to 1.76) | 0.8 (0.63 to 1.02) |
| Missing/Not known | 1.42 (0.84 to 2.39) | 1.06 (0.82 to 1.38) |
| Other | 1.47 (0.75 to 2.88) | 0.89 (0.61 to 1.29) |
|  |  |  |
| **Surgical procedure category (vs. cardiac)** | | |
| Bone | 0.55 (0.13 to 2.35) | 0.71 (0.52 to 0.98) |
| Breast | 0.06 (0.01 to 0.45) | 0.31 (0.04 to 2.53) |
| Cerebrovascular | 0 (0 to 3.1391414647971E+156) | 0.92 (0.28 to 3.02) |
| Ear | 0 (0 to 2.75147101962783E+33) | 0 (0 to 9.02145571327597E+69) |
| Endocrine | 1.02 (0.3 to 3.5) | 2.3 (0.88 to 5.98) |
| Female LGU | 0 (0 to 2.82590198392492E+46) | 0.8 (0.17 to 3.69) |
| Female UGU | 0.06 (0.01 to 0.46) | 0 (0 to 149377138165961000) |
| HPB | 0.64 (0.27 to 1.51) | 1.72 (1.05 to 2.81) |
| Joint | 0.1 (0.02 to 0.41) | 0.88 (0.61 to 1.27) |
| Lower GI | 0.45 (0.23 to 0.9) | 0.87 (0.63 to 1.19) |
| Major Vessel | 1.12 (0.49 to 2.6) | 1.25 (0.84 to 1.87) |
| Male GU | 0.96 (0.22 to 4.19) | 1.25 (0.47 to 3.34) |
| Muscle | 0 (0 to >20) | 0.74 (0.22 to 2.43) |
| Nasal | 0.62 (0.14 to 2.67) | 1.59 (0.81 to 3.14) |
| Neuro | 0.73 (0.3 to 1.83) | 2.48 (1.78 to 3.46) |
| Ocular | 0.33 (0.17 to 0.66) | 0.52 (0.12 to 2.23) |
| Oral | 0.45 (0.15 to 1.34) | 0.6 (0.27 to 1.37) |
| Orthopaedics | 1.24 (0.16 to 9.39) | 0 (0 to >20) |
| Other | 1.33 (0.71 to 2.46) | 2.81 (1.78 to 4.42) |
| Pharynx | 0.72 (0.16 to 3.14) | 1.48 (0.5 to 4.41) |
| Skin | 0.64 (0.32 to 1.27) | 0.65 (0.46 to 0.92) |
| Skull & Spine | 0.07 (0.01 to 0.51) | 1.28 (0.79 to 2.07) |
| Thoracic | 1.49 (0.83 to 2.69) | 3.23 (2.47 to 4.23) |
| Upper GI | 1.71 (0.78 to 3.74) | 2.42 (1.62 to 3.63) |
| Urological | 0.2 (0.08 to 0.48) | 1.21 (0.86 to 1.72) |
| Vascular | 1.03 (0.53 to 1.98) | 1.77 (1.34 to 2.34) |
| **Smoking status (vs. Never)** | | |
| Current | 2.1 (1.39 to 3.16) | 1.37 (1.13 to 1.67) |
| Former | 1.41 (0.99 to 2.02) | 0.94 (0.79 to 1.12) |
| Unknown | 1.11 (0.48 to 2.57) | 0.9 (0.62 to 1.32) |

**Supplementary table 4. Summary of logistic regression models used in Elective & Emergency surgical settings to risk-stratify the cohort.** Data are presented as odds ratios with associated 95% confidence intervals. Surgical procedure category is based on the anatomical location of the index procedure code. Index of multiple deprivation is national quintiles.

|  | **Number**  **(%)** | **Total healthcare encounters**  **(days per patient year)** | | | **Death by two years**  **(%)** | **Median DAH-90 (IQR)** |
| --- | --- | --- | --- | --- | --- | --- |
|  |  | Before | After | Rate ratio |  |  |
| **Major surgery** | 19,236 (27.5%) | 13.9 | 22.4 | 1.60 (1.60-1.61)* | 1740 (9.0%) | 84 (77 to 87) |
| **Non-major surgery** | 50,785 (72.5%) | 12.6 | 16.1 | 1.27 (1.27-1.27)* | 2745 (5.4%) | 89 (86 to 90) |

**Supplementary table 5. Number of patients, number of days in contact before and after surgery, and rate of death by two years, stratified by the presence of a code indicating major surgery.** Data are presented as encounters per patient year unless otherwise stated. Number is the total number and percentage of the whole cohort, death by two years is the number of patients dying within 2 years of index surgical procedure and associated percentage. DAH-90; Days alive at home within 90-days reported as median with interquartile range.

| **Variable** | **Incidence rate ratio** |
| --- | --- |
| Intercept | 0.01 (0.01 to 0.01) |
| Age 18-39* | 1.41 (1.37 to 1.45) |
| Age 39-58* | 2.33 (2.2 to 2.47) |
| Age >58* | 2.13 (2.04 to 2.22) |
| Inpatient vs. Daycase | 1.34 (1.32 to 1.36) |
| Emergency vs. Elective | 1.58 (1.56 to 1.61) |
| Male vs. Female | 0.92 (0.91 to 0.93) |
| *Days of healthcare use 2 years before surgery* | |
| 0 - 5 | 2.03 (1.96 to 2.1) |
| 5.1 - 8.5 | 2.88 (2.77 to 2.99) |
| 8.6 - 12.5 | 15.34 (13.9 to 16.94) |
| 12.5 - 19.9 | 10.27 (9.33 to 11.31) |
| >19.9 | 2.01 (1.85 to 2.19) |
| *Charlson comorbidity index domains* | |
| Cardia Failure | 1.28 (1.24 to 1.31) |
| Myocardial Infarction | 0.98 (0.95 to 1.01) |
| Peripheral vascular disease | 1.28 (1.24 to 1.32) |
| Stroke | 1.19 (1.15 to 1.22) |
| Dementia | 1.41 (1.35 to 1.47) |
| Respiratory disease | 1.1 (1.08 to 1.11) |
| Rheumatological disease | 1.13 (1.09 to 1.18) |
| Liver disease | 1.3 (1.25 to 1.35) |
| Diabetes Mellitus | 1.1 (1.09 to 1.12) |
| Hemiplegia | 1.17 (1.1 to 1.25) |
| Chronic kidney disease | 1.11 (1.09 to 1.13) |
| Cancer | 2 (1.96 to 2.04) |
| Human Immunodeficiency virus | 1.14 (1.05 to 1.25) |
| *Procedure category (vs. Cardiac)* | |
| Bone | 1.3 (1.26 to 1.35) |
| Breast | 1.05 (1 to 1.1) |
| Cerebrovascular | 1.18 (1.04 to 1.35) |
| Ear | 1.01 (0.96 to 1.06) |
| Endocrine | 1.06 (1 to 1.14) |
| Female LGU | 0.95 (0.9 to 1.02) |
| Female UGU | 0.96 (0.94 to 0.99) |
| HPB | 1.06 (1.02 to 1.11) |
| Joint | 1.2 (1.17 to 1.24) |
| Lower GI | 1.03 (1 to 1.06) |
| Major Vessel | 1.32 (1.25 to 1.4) |
| Male GU | 0.75 (0.71 to 0.79) |
| Muscle | 1.23 (1.18 to 1.28) |
| Nasal | 0.99 (0.95 to 1.04) |
| Neuro | 1.42 (1.37 to 1.47) |
| Ocular | 1.04 (1.01 to 1.07) |
| Oral | 0.89 (0.86 to 0.92) |
| Orthopaedics | 1.24 (1.13 to 1.37) |
| Other | 1.45 (1.38 to 1.52) |
| Pharynx | 1.04 (0.98 to 1.1) |
| Skin | 1.06 (1.03 to 1.09) |
| Skull&Spine | 1.17 (1.13 to 1.21) |
| Thoracic | 1.61 (1.54 to 1.68) |
| Upper GI | 1.53 (1.44 to 1.63) |
| Urological | 1.09 (1.05 to 1.12) |
| Vascular | 1.14 (1.1 to 1.17) |

**Supplementary table 6. Summary of negative binomial model demonstrating the association between preoperative features and subsequent healthcare use.**

**Supplementary figure 1. Proportion of patient days in contact with healthcare in the two years before and after surgery (solid line), and cumulative rate of death (dashed line), among patients undergoing elective surgery.** Each point represents the proportion of patient days spent in contact with healthcare during the 7-day window, with associated 95% confidence intervals. All patients contact healthcare on day 0 (i.e. the date of surgery), and this was excluded from modelling to prevent distortion

**Supplementary figure 2.** **Proportion of patient days in contact with healthcare in the two years before and after surgery (solid line), and cumulative rate of death (dashed line), stratified by risk group undergoing emergency surgery.** Data aggregated by 7-day windows, each of which has 95% confidence intervals. Each point represents the proportion of patient days spent in contact with healthcare during the 7-day window. All patients contact healthcare on day 0 (i.e. the date of surgery), and this was excluded from modelling to prevent distortion. The number in brackets indicates the proportion of patients in each risk group.


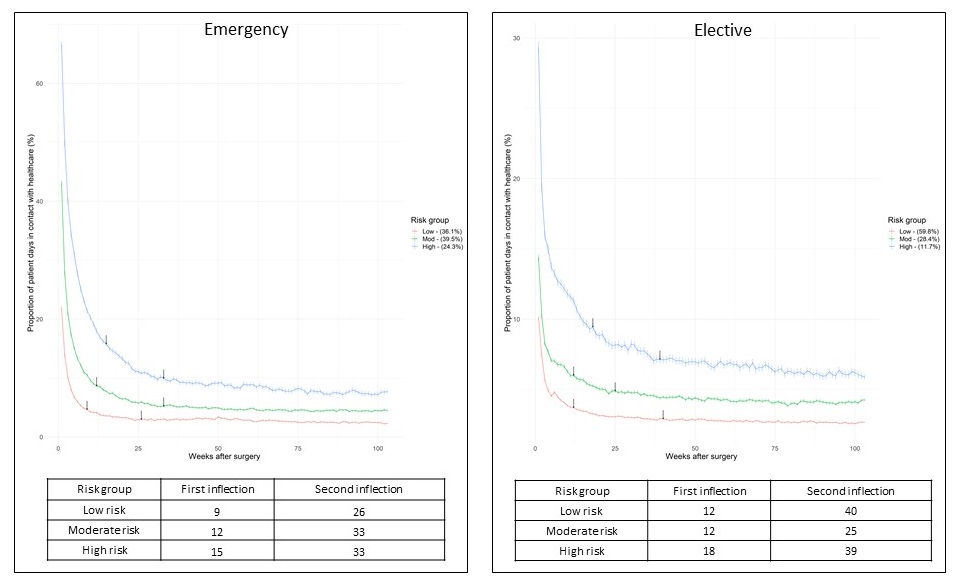
**Supplementary figure 3. Proportion of patient days in contact with healthcare following surgery, presented for each risk group within emergency and elective surgery.**  Arrows indicate inflection points identified by unit invariant knee modelling. Time to inflection in table below is listed in weeks following surgery.

**
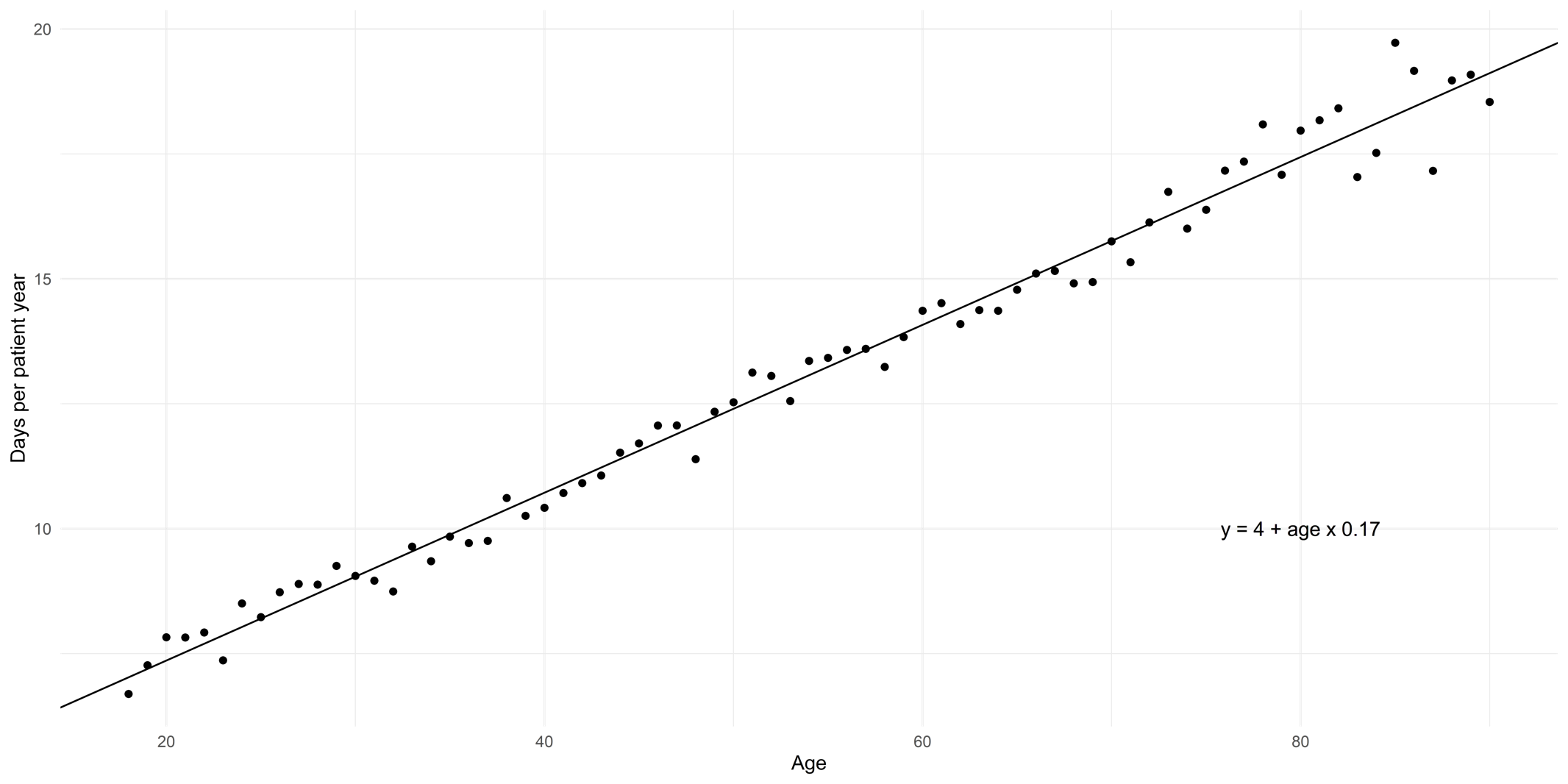
**

**Supplementary figure 4. Increase in encounters as age increases.** Data estimated using a linear regression model.
